# Association between Age at Diagnosis of Graves' Disease and Variants in Genes Involved in Immune Response

**DOI:** 10.1371/journal.pone.0059349

**Published:** 2013-03-27

**Authors:** Beata Jurecka-Lubieniecka, Rafal Ploski, Dorota Kula, Aleksandra Krol, Tomasz Bednarczuk, Zofia Kolosza, Andrzej Tukiendorf, Sylwia Szpak-Ulczok, Anita Stanjek-Cichoracka, Joanna Polanska, Barbara Jarzab

**Affiliations:** 1 Department of Nuclear, Medicine and Endocrine Oncology, Maria Sklodowska-Curie Memorial Cancer, Center and Institute of Oncology, Gliwice Branch, Gliwice, Poland; 2 Department of Forensic Medicine, Medical University of Warsaw, Warsaw, Poland; 3 Department of Isotopic Diagnostic and Radiopharmaceutical, Medical University of Silesia, Katowice, Poland; 4 Institute of Automatic Control, Silesian Technical University, Gliwice, Poland; University of Cagliari, Italy

## Abstract

**Background:**

Graves' disease (GD) is a complex disease in which genetic predisposition is modified by environmental factors. The aim of the study was to examine the association between genetic variants in genes encoding proteins involved in immune response and the age at diagnosis of GD.

**Methods:**

735 GD patients and 1216 healthy controls from Poland were included into the study. Eight genetic variants in the *HLA-DRB1*, *TNF*, *CTLA4*, *CD40*, *NFKb*, *PTPN22*, *IL4* and *IL10* genes were genotyped. Patients were stratified by the age at diagnosis of GD and the association with genotype was analysed.

**Results:**

Polymorphism in the HLA-DRB1, TNF and CTLA4 genes were associated with GD. The carriers of the HLA DRB1*03 allele were more frequent in patients with age at GD diagnosis ≤30 years than in patients with older age at GD diagnosis.

**Conclusions:**

HLADRB1*03 allele is associated with young age at diagnosis of Graves' disease in polish population.

## Introduction

Graves' disease (GD) is an autoimmune disorder. Genetic predisposition modified by environmental factors is responsible for the pathogenesis if GD. Several genetic variants associated with GD were found, but none of them was introduced into clinical diagnostics [1]. An inherited predisposition to autoimmune thyroid diseases, including GD, currently involves about 20 identified genetic polymorphisms, including both thyroid-specific genes and those regulating the autoimmune response. Analyzed variants showed little impact on the development of GD with OR = 1,2–1,5 [2]. Interaction among genetic variants may increase the risk of GD disease [3]. Environment has a proven impact on the GD risk [4]–[5]. The impact of genetic factors may be higher than environmental factors in patients with young age at GD diagnosis [6]. GD is more frequent in the children of parents with young age at GD diagnosis [7]–[8]. Disease phenotype is also different between patients with young age at GD diagnosis than in patients with older age at GD diagnosis [9]–[12].

The aim of the study was to examine the association between genetic variants in genes encoding proteins involved in immune response and the age at GD diagnosis in polish population. Genetic variants in following genes were analyzed: *HLADRB1, CTLA4, PTPN22*, *CD40, TNFα*, *NFkB, IL4 and IL10.* Proteins coded by these genes are involved in development of autoimmune diseases [13]–[15].

## Materials and Methods

### Subjects

735 Polish patients diagnosed with GD and 1,216 healthy control subjects were included into the study. Patients with GD were consecutively recruited in the Department of Nuclear Medicine and Endocrine Oncology, Center of Oncology in Gliwice, Poland (n = 337) and in the Department of Endocrinology, Medical University of Warsaw, Poland (n = 398). All individuals were Caucasian. The clinical characteristics of GD patients is presented in Table 1. The diagnosis of GD was based on clinical and biochemical symptoms of hyperthyroidism and detectable TSHR autoantibodies (Brahms). The severity of ophthalmopathy was assessed according to the NOSPECS classification. The study was approved by the ethics committees of both participating institutions and written informed consent was obtained from all participants. The control group comprised 1216 subjects. The Gliwice control group (n = 344) consisted of healthy volunteers with euthyroid function, normal thyroid ultrasound image, and with no family history of GD. The Warsaw control group (n = 872) included anonymous, unrelated adults collected in the Regional Blood Transfusion Center.

**Table 1:** Clinical characteristics of patients with Graves' disease.

	N = 735
Gender [female/male]	588/147
Age at GD diagnosis [mean±SD, years]	41,3±14,27
Ophthalmopathy (NOSPECs≥2) [n (%)]	278 (39,6%)
Tobacco smokers [n [%)]	326 (48,2%)
Duration of GD after being diagnosed by physician [mean±SD, years]	2,63±0,69

### Methods

Genomic DNA was extracted from whole blood according to standard protocols by salt protein precipitation. HLA-DRB1 genotyping in Gliwice was performed using sequence-specific oligonucleotides (SSO, Innolipa HLA-DRB1, Innogenetics) and sequence-specific primers (MSSP Class II DRB Only, One Lambda, Dynal All Set SSP DR test, Dynal Biotech) [16] and by Dynal All Set SSP Dr test (Dynal Biotech) in Warsaw [17]. The *TNF, CTLA-4, NFKB, CD40,* and *PTPN22 genetic variants* were genotyped by the PCR-RFLP method (Table 2) [16], [18]–[20]. PCR products were digested by appropriate restriction endonuclease and digestion products were separated on the 3% agarose gel.

**Table 2:** Methods of genotyping of analyzed polymorphism.

Gene	SNP	Location	Method	PCR Primer F 5′-3′ Primer R 5′-3′	RFLP enzyme
**HLA-DRB1**	HLA-DRB1*03	exon 2	PCR-SSP/PCR-SSO	–	–
**TNF**	G(-308)A rs1800629	promoter	PCR-RFLP	F:AGGCAATAGGTTTTGAGGGCCAT R:TCCTCCCTGCTCCGATTCCG	*Nco*I
**CTLA-4**	A(49)G rs231775/rs57563726	Codon 1, exon 3	PCR-RFLP	F:CCAAGTCTCCACTTAGTTATCCR:CCTCCATCTTCATGCTCC	*Bst71*I
**NFKB**	94ATTG ins/del rs28362491	promoter	PCR-RFLP	F:TTTAATCTGTGAAGAGATGTGAATG R:CTCTGGCTTCCTAGCAGGG	*Van91*I
**CD40**	C(−1)T rs1883832	Kozak sequence	PCR-RFLP	F:CCTCTTCCCCGAAGTCTTCCR:GAAACTCCTGCGCGGTGAAT	*Sty*I
**PTPN22**	C(1858)T rs2476601	codon 620,exon 14	PCR-RFLP	F:TCACCAGCTTCCTCAACCACA R:GATAATGTTGCTTCAACGGAATTT	*Xcm*I
**IL10**	A(−1082)G rs1800896	promoter	ARMS-PCR	FA:ACTACTAAGACTTCTTTGGGAA FG:CTACTAAGGCTTCTTTGGGAGR:AGAAGTCCTGATGTCACTGC	-
**IL4**	C(−589)T rs2243250	promoter	PCR-RFLP	F:CACCTAAACTTGGGAGAACATTG R:GGAAAGATAGAGTAATATCA	*Ava*II

### Statistical Analysis

Statistical analysis was performed using the statistical program STATA12.0. The distribution of genotypes and alleles in both groups was compared using the chi-squared test or the Fisher's Exact Test. Bonferroni correction was applied for multiple comparisons. Hardy-Weinberg equilibrium was analyzed.

Due to the non-random allele association, the linkage disequilibrium ratios D' were estimated. We performed multivariate logistic regression taking into the model HLA and TNF. To estimate the haplotypes effect on the disease risk we performed analysis using “haplologit” command (STATA). The EM algorithm was used to calculate the haplotype frequency of two polymorphisms (HLADRB1*03, −308A TNF), localized on chromosome 6.

## Results

### Allele Frequency Analysis

The genotype frequency in control group was in Hardy-Weinberg equilibrium. HLADRB1*03, −308A TNF and 49G CTLA4 genetic variants had different allele frequency in GD patients than in controls (Table 3). GD patients and healthy controls did not differ when allele frequency of genetic variants in the *CD40, NFkB*, *PTPN22, IL4 and IL10* genes was analyzed (Table 3). The frequency of the DRB1*03 allele in the GD patients was higher than the control group (23% vs. 10%, p = 1,76*10^−6^) (Table 3). Similarly the −308A allele of the *TNF* gene and the 49G allele of the *CTLA4* gene occurred significantly more frequently in GD patients than in the control group (25% vs. 14%, p = 7,33*10^−8^ and 50% vs. 40%, p = 6,21*10^−4^ respectively) (Table 3).

**Table 3:** Allele frequency in patients with Graves' disease (GD)and control group.

Gene	SNP	Allele	GD n (%)	Control n (%)	p*
**HLA-DRB1**	HLA-DRB1*03	DR3−	650 (77)	359 (90)	6.7×10^−7^
		DR3+	194 (23)	41 (10)	
**TNF**	G(−308)A rs1800629	G	829 (75)	589 (86)	2.79×10^−8^
		A	281 (25)	93 (14)	
**CTLA-4**	A(49)G rs231775	A	620 (50)	411 (60)	2.36×10^−4^
		G	620 (50)	275 (40)	
**NFKB**	94ATTG ins/del rs28362491	94ATTG+	714 (55)	237 (61)	ns
		94ATTG−	582 (45)	151 (39)	
**CD40**	C(−1)T rs1883832	C	226 (22)	100 (25)	ns
		T	802 (78)	300 (75)	
**PTPN22**	C(1858)T rs2476601	C	328 (83)	336 (85)	ns
		T	68 (17)	60 (15)	
**IL-10**	A(−1082)G rs1800896	A	257 (43)	262 (42)	ns
		G	343 (57)	360 (58)	
**IL-4**	C(−589)T rs2070874	C	331 (83)	293 (81)	ns
		T	69 (17)	67 (19)	

* with Bonferroni correction.

### Genotype Frequency Analysis

We observed significantly more frequent occurrences of at least one DRB1*03 allele in the GD patients compared to healthy subjects (OR 3,41; 95%CI 2,24–5,26) (Table 4).

**Table 4:** Genotypes of 4 polymorphisms with significant differences in allele frequencies between patients with Graves' disease (GD) and control group.

Gene	SNP	Genotype	GD n(%)	Control n(%)	p/OR/95% CI
HLADRB1	HLA-DRB1*03	DRB3−/−	238 (56)	163 (81)	
		DRB3−/+	174 (41)	33 (17)	
		DRB3+/+	10 (3)	4 (2)	
		DRB3−/+ and +/+			1,75*10^−8^
		vs. DRB3−/−			3,41 (2,24–5,26)
TNF	G(−308)A	GG	299 (54)	259 (76)	
		GA	231 (42)	71 (21)	
		AA	25 (4)	11 (3)	
		GA+AA vs. GG			5,78*10^−10^ 2,70 (1,99–3,70)
CTLA4	A49G	AA	159 (26)	126 (37)	
		AG	302 (49)	156 (46)	
		GG	159 (25)	58 (17)	
		AG+GG vs. AA			0,03 1,68 (1,25–2,26)

Analysis of the *TNF* gene polymorphism revealed significantly higher incidence of AG and AA genotypes in GD patients compared to controls, with increased risk for carriers of at least one −308A allele (OR = 2,70; 95%CI 1,99–3,70) (Table 4).

In case of the *CTLA4* gene genotypes showed more frequent presence of GG homozygotes among patients compared to controls and increased GD risk in G allele carriers (OR 1,67, 95% CI 1,19–2,39) (Table 4).

### Haplotype Frequency Analysis

Since *TNF* gene is located within the *HLA* genes we examined the haplotypes containing alleles HLADRB1*03 and −308A of *TNF.* A strong linkage disequilibrium among genetic variants in the TNF and HLADRB1 genes was observed (D’>0,8). The most common haplotype in GD patients was the one containing all two risk alleles. It was present in 20% of patients, which was associated with a significantly elevated GD risk (OR = 3,16, p = 1,94*10^−11^) relative to 9% in healthy people.

### Relationship between the HLA and the CTLA4 Susceptibility Genes

The interaction of HLADRB1*03 and A(49)G CTLA4 polymorphisms was tested to check whether they exert any additional influence on GD development. They were found to act independently: the combined OR for the two alleles was 1,01.

### Genotype-phenotype Correlation

The genotype containing at least one DRB1*03 allele was associated with younger age at GD diagnosis (Table 5). No association was observed between DRB1*03 allele and duration of pharmacotherapy, GD relapse rate, number of radioiodine treatment courses, thyroid receptor (TR) Ab level and gender. The association between the HLADRB1*03 allele and age at GD diagnosis was confirmed in multivariable analysis. Graves’ ophthalmopathy of NOSPECS≥2 was diagnosed in 278 (39.6%) patients (Table 1). Carriers of the HLADRB1*03 allele were significantly more common in young patients with Graves’ ophthalmopathy (p = 0.001) (Table 6).

**Table 5:** HLA-DRB1-03 genotypes in patients with Graves' disease (GD) stratified by the age at GD diagnosis.

SNP	Genotype	GD≤30 n (%)	GD>30 n (%)	OR (95% CI)/p
HLA-DRB1*03	DR3−/−	42 (42)	196 (61)	Reference
	DR3−/+	56 (55)	118 (37)	2,21(1,36–3,61) p<0,001
	DR3+/+	3 (3)	7 (2)	2,00 (0,31–9,18) ns
	DR3−/+ and +/+	59 (58)	125 (39)	2,20 (1,36–3,57) p<0,001

**Table 6:** Distribution of HLA DRB1*03 and TNF polymorphisms in GD patients stratified by the diagnosis of Graves’ ophthalmopathy.

SNP	Genotype	NOSPECS	GD ≤30 n (%)	GD >30 n (%)	*p*
HLA-DRB1*03	DR3−/−	2+	15 (35,7)	112 (63,6)	**0.001**
	DR3−/+ and +/+		27 (64,3)	64 (36,4)	
	DR3−/−	0–1	26 (44,8)	84 (57,9)	Ns 0.062
	DR3−/+ and +/+		32 (55,2)	61 (42,1)	
TNF	GG	2+	18 (37,5)	132 (55,7)	**0.016**
	AA and AG		30 (62,5)	105 (44,3)	
	GG	0–1	29 (44,6)	119 (58,3)	**0.037**
	AA and AG		36 (55,4)	85 (41,7)	

## Discussion

We confirmed previous observations, that genetic variants in HLA-DRB1, TNF and CLTA-4 genes are associated with GD. We found an association between the HLA-DRB1-03 polymorphism and young age at GD diagnosis. A similar association between genetic markers and age at diagnosis was reported in type 1 diabetes [21], rheumatoid arthritis [22] and multiple sclerosis [23]. GD was diagnosed at younger age in patients with family history of this disease [9], [24].

We found a significant association between HLA DRB1*03 and the age at GD diagnosis when study population was stratified by the age of 30 years at GF diagnosis. Patients with age ≤30 years at GD diagnosis had higher frequency of the HLADRB1*03 allele. Genotypes containing the HLADRB1*03 allele occurred almost twice as much in patients at younger age at GD diagnosis. In our research, young carriers of the DR3 polymorphism were more common than older ones, both in family and sporadic GD. These results suggest the existence of different genetic conditions for the development of GD in young people. It can be assumed that earlier recognition of the disease in case of young patients with positive family history (FH) results from the increased alertness of ill parents. However, a violent course of disease in case of young people with distinct clinical symptoms forces to quick medical consultation regardless to FH [9], [24]. Young carriers of HLADRB1*03 allele are likely to be more susceptible to GD, despite a shorter exposure to environmental factors. HLADRB1*03 is necessary for induction of the immune response. The effect of HLA on the age at GD diagnosis can be explained by the molecular mechanism of initiation of the autoimmune processes involving the identification of an antigen bound to HLA molecule by T cells [25]. It has been suggested that the presence of the HLADRB1 allele with arginine at position 74 of the B1 chain changes the shape of the antigen-binding groove, allowing effective presentation of the TSH receptor peptide in the Caucasian population [26].

This is the first study in population of Poland showing association between HLADRB1*03 and age at GD diagnosis. There are only few studies which showed the association of this polymorphism with younger age at GD diagnosis. In the Caucasian population, the association of *HLADRB1* with GD diagnosis at a younger age has previously been shown in two studies. Lavard et al. affirmed that the presence of the HLADRB1*0301 allele resulted in a higher relative risk of GD among ninety Danish young patients [27]. Farid et al. observed an association between the HLADRB1*03 allele and eighty-three younger GD patients, taking the age of 30 as a cut-off line [28]. The association of other *HLA* alleles with the age at GD diagnosis was also demonstrated in other ethnic groups - in the Chinese [29] and Japanese population [30]. The value of our study is a large well characterized population of GD patients from polish population. The carriers of the HLA DRB1*03 allele who are predisposed to earlier onset of GD may require further diagnostics for other autoimmune diseases.

We observed a higher frequency of the HLADRB1*03 allele in young patients with Graves’ ophthalmopathy. The results of genetic tests assessing the effect of *HLADR3* on the development of ophthalmopathy in whole, age-irrespective groups of patients are incompatible [31]–[35]. On the other hand, there are no reports concerning the role of HLA in the development of ophthalmopathy in young GD patients, despite the fact that the clinical course of the disease is different in different age groups [36]-[39]. Objective difficulties in explaining the genetic background of ophthalmopathy in young patients are due to its significantly lower incidence in this age group, while in adults those difficulties are associated with the impact of environmental factors, especially smoking [40]. Other factors interfering with the research include clinical course of ophthalmopathy, and the time of its occurrence in relation to hyperthyroidism [41], which requires long-term observation of the patient.

While analysing the interaction between HLADRB1*03 and CTLA4 genetic variants we found no additive effect on the risk of GD. The impact of HLADRB1*03 [28], [42] and 49G of CTLA4 [43]-[44] on GD development was proven in many studies. Similarly as in case of our research, other authors reported only a weak association between GD and genetic variants in the CTLA4 gene [45].

We did not observed an HLA-independent effect of −308A TNF variant on the risk of GD. This is consistent with previous reports [46]–[47]. Following multivariate logistic regression, the statistically significant effect of HLA has been established, only; the effect of polymorphism TNF was statistically insignificant. This suggests the leading effect of the HLA polymorphism on GD.

We were not able to confirm the association between PTPN22 and GD, what was reported by other research groups. However we observed a higher frequency of the risk T allele in GD patients than in control group, the differences was not significant. This may be caused by a potential dissimilarity among analysed populations [48].

### Conclusion
